# Pathogenic helminths in the past: Much ado about nothing

**DOI:** 10.12688/f1000research.11752.3

**Published:** 2017-08-11

**Authors:** Christian Mulder

**Affiliations:** 1 National Institute for Public Health and the Environment (RIVM), Bilthoven, 3721, Netherlands

**Keywords:** Human diseases; Endoparasitic nematodes; Roman settlements; Fossil eggs.

## Abstract

Despite a long tradition on the extent to which Romanisation has improved human health, some recent studies suggest that Romanisation in general, and Roman sanitation in particular, may not have made people any healthier, given that in Roman times gastrointestinal parasites were apparently widespread, whilst in the present day such parasites rarely cause diseases. Unfortunately, this novel claim neglects the empirical evidence that worldwide infections in over 1.5 billion people are caused by ubiquitous foodborne nematodes. Therefore, many may wonder if fossil remains of soil-transmitted helminths have been reported in ancient sanitation infrastructures. Beneficial access to improved sanitation should always be prioritized, hence how can historical sanitation efforts have ever been harmful? In this short article, a strong plea for caution is given, asking for an augmented nematological record and showing that there is not any evidence against Roman sanitation, neither in the past nor in the present.

In her *Nature* feature, Chelsea Wald ^1^ reviewed some of the conclusions by Piers D. Mitchell ^2^ and describes the fascinating rise of latrines in Mesopotamia, Greece and the Roman Empire. Both authors tried to point out that most of these sanitation facilities were not doing much for the residents’ health, despite the idea that sophisticated plumbing systems, like those of ancient Rome, may have acted as a kind of control that could benefit even the poor. This debated interpretation was based on the fact that human hosts mainly acquire infective nematodes via the faecal–oral route through the soil, although unembryonated eggs can remain viable in the soil for 15 years. Helminth preservation seems to be the highest in moist anaerobic environments like latrines ^3^; therefore, even Roman latrines, with continuous flushing and related sediments (coprolites), can become valuable for the reconstruction of past gastrointestinal infections, if evaluated correctly.

As a matter of fact, water purification will always be one of the most intriguing examples of how public health and societal health are interwoven. Amazing examples come from Roman history, where water and wastewater systems rapidly became pillars for European civilisation. The large-scale introduction by Romans of fountains into or near public buildings, together with closed aqueducts, can be seen as the very first Water Safety Plan. Interestingly, archaeologists somehow seem to be ideologically motivated to conceptualize diseases and outbreaks in Roman times, despite the thin palynological record from ancient sanitation infrastructures around the Mediterranean Sea ^1, 2 ^ ( Figure 1).

**Figure 1.**
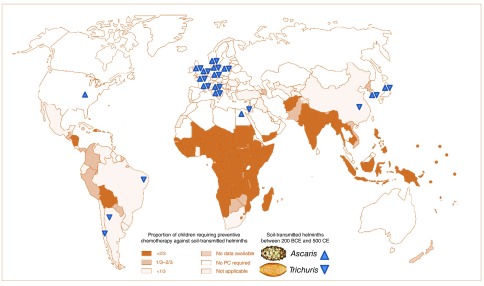
Multiple geographical and historical investigation bias between ancient settlements claimed to be infected in the past, and children’s helminthiases in modern countries. Background map implemented with palaeoparasitological records of roundworms ( *Ascaris*) and whipworms ( *Trichuris*) recovered from a global selection of archaeological sites built between 200 BCE and 500 CE (Common Era) ^2, 4– 8^. The background map has been adapted from World Health Organization, program on Control of Neglected Tropical Diseases ( gamapserver.who.int/mapLibrary/Files/Maps/STH_2011_global.png).

Many pathogens are reported in ancient latrines because they are intrinsically correlated to human settlements, and not to sanitation infrastructures themselves, which are supposed to reduce the risk of contact with outbreak sources. On one hand, it is true that fossil remains of roundworms, whipworms and hookworms (collectively referred to as soil-transmitted helminths) have been reported from ancient sanitation infrastructures. On the other hand, Romans were fully aware of the importance of clean water and efficient sanitation systems. Already during the short reign of Nerva in Rome (96 – 98 CE), Frontinus decided that water from different sources had to be kept separate: clean water was reserved for potable use, intermediate quality water was used for recreation and only poor quality water was sent for irrigation. Thanks to sophisticated hydraulic systems for aqueducts, cisterns, pipes, therms, baths, fountains and latrines, the capital of the Roman Empire became famous as *Roma regina aquarum*. Due to the greatest care provided to their waters for the health and security of the capital and later of the other cities, these neglected latrines became an open archaeological window not so much on Roman sanitation, but on our civilisation as a whole. Fossilized helminth eggs in dung sediments from latrines are very peculiar tools to reconstruct migration routes, trades, animal domestication, diets, past outbreaks and even urban catastrophes ^4– 6^.

Nematodes are the most frequently occurring invertebrates. These primitive soil organisms occupy diverse trophic levels in ecological networks and can act either as antagonists for soil-borne pests or be pathogens themselves. It can be dangerous to suggest that sanitation may not have made people any healthier, as humans can also get infected with soil nematodes by ingesting unclean vegetables or by contact with infected domestic animals. Along the aforementioned faecal–oral route, behavioral and allometric factors have been put forward in existing literature ^9^, being the host-related factors linked to human size prominent. According to host–parasite regression models for mammals ^9^ and assuming on average one adult body weight of 62.0 kg (corresponding to a volume of 61,400 cc), each infected human might contain up to 12,300 helminths. 

Hence, it is not surprising to find helminths in sanitation systems of ancient settlements, especially if only the palaeoparasitological data for sites at which these pathogens were detected are gathered together. For instance, archaeological records of common-source outbreaks can be collated to support the idea that sanitation facilities historically linked to Romanisation have widespread helminths, although these cosmopolitan endoparasites are well-known to occur during Roman times even around the Pacific Ocean, including the New World in pre-colonial times ^5, 7, 8, 10^ ( Figure 1). Thus, we have to realize that there would be many more helminth eggs in ancient sanitation facilities if these facilities had not been there in the past. Surprisingly, archaeologists like to invert this basic framework, and suggestive interpretation may be worse than no interpretation at all. 

But even if such pathogens are identified, it remains challenging either to exclude false parasitism (incidental presence in human faeces of eggs resulting from the consumption of an infected animal ^7, 11^) or to determine with certainty human outbreaks (helminth eggs might demonstrate their human origin by some circumstantial evidence only ^3, 11^). Allometric rules that express parasites and non-infected animals per square meter ^12^, in tandem with the several possible contamination pathways, will always lead to diseases with a high global burden ^13^. Moreover, a parasitic occurrence can also be related to open water contamination, for instance from livestock grazing in upland areas causing outbreaks downstreams. This has nothing to do with any sanitation structure, as these parasites are by far common and overdispersed ^14– 16^: hot spots examples of faecal contamination, as shown by highly aggregated *Ascaris* or *Trichuris* eggs, are in fact well-known in archaeology and palaeoecology ^11, 17, 18^.

Omitting such a relevant weight of evidence in any comparison between archaeological excavations will introduce *de facto* a strong bias towards false-positive results into palaeoecological meta-analyses. In the future, to avoid interesting, but geographically misleading or even statistically speculative conclusions, one of the most intriguing hypotheses that will arise might be the investigation by microscopy of soils from archaeological sites associated either with one sanitation infrastructure or without that sanitation. In the case of Roman sanitation, due to the Hadrian’s Wall bordering the northern part of the Roman Empire with all its social infrastructures, including latrines, England (entirely inside the Wall during Roman times) and Scotland (outside the Wall during Roman times) can together provide the perfect study area. Again, it seems necessary to emphasize that the Romans created an original system of public sanitation and water distribution on a scale not seen before simply because they linked as first individual hygiene to public health. As it is all too easy to spot patterns in randomly collected data, objective empirical evidence that correlations are real demands a well-designed survey to avoid any suspicion.

There are 2.5 billion people still living on Earth without improved sanitation facilities and an ensured availability of sanitation worldwide is one of the core United Nations targets to end poverty in 2030 ( http://www.un.org/sustainabledevelopment/sustainable-development-goals). Therefore a correct data mining of all nematological records ^19^ combined with objective interpretation of, probably thin, circumstantial evidence will require great care, as the conclusion shall have implications on ongoing global control programs relating to helminthiases. On the other hand, as the taxonomic status of *Ascaris* is contentious ^20^, palaeoecological evidence from archaeological sites in synergy with present-day molecular ecology can become an unexplored avenue to improve current control programs.
