# Supplementary material for: Pathogenic helminths in the past: Much ado about nothing
Source: F1000Res. 2017 Aug 11;6:852. Originally published 2017 Jun 8. [Version 3] doi: 10.12688/f1000research.11752.3 (PMC5580415; doi:10.12688/f1000research.11752.3)
Supplement: Peer review history

# Peer review: Referee response for version 3

**DOI:** 10.5256/f1000research.13422.r24946

**Published:** 2017-08-22

**Authors:** Cornelis van Tilburg

**Affiliations:** 1 Centre for the Arts in Society, Leiden University, Leiden, Netherlands

After his revisions, I agree with the revised article of Christian Mulder.

I have read this submission. I believe that I have an appropriate level of expertise to confirm that it is of an acceptable scientific standard.

# Peer review: Referee response for version 2

**DOI:** 10.5256/f1000research.13196.r24554

**Published:** 2017-08-08

**Authors:** Cornelis van Tilburg

**Affiliations:** 1 Centre for the Arts in Society, Leiden University, Leiden, Netherlands

In Mulder’s article “Pathogenic helminths in the past: Much ado about nothing”, Chelsea Wald and Piers D. Mitchell state that sanitation did not improve people’s health significantly, despite its sophisticated technique. In coprolites and remains of toilets throughout the Roman Empire the presence of helminth’s eggs of *Ascaris* and *Trichuris* was widespread, testifying to the unhealthiness of the environment.

 Mulder states that helminths arrive in the faeces deposit (dung heap or sewer system) through the oral-faecal route; people are infected by eating infected food and drinking infected water, so that there is no immediate connection between sanitation and ‘eating’ helminth’s eggs. Mitchell correctly observes that a concentration of faeces is a breeding-place for parasites and eggs. Faeces was used as a fertilizer in the countryside, so that helminth’s eggs were spread.

 Comparing ancient sanitation with modern sanitation is not without risk. Mulder’s statement ‘The large-scale introduction by Romans of fountains into or near public buildings, together with closed aqueducts, can be seen as the very first Water Safety Plan’ (second paragraph, ‘As a matter … Sea’) is disputable. The aqueducts were closed, indeed, but this construction served to prevent pollution (e.g. by pieces of wood) or even poisoning ^1^. Moreover, ancient ‘safe water’ differs from modern ‘safe water’ ^2^. Nowadays, ‘safe water’ is water without pollution of by parasites, chemicals, etc. In Antiquity, ‘safe water’ was clear, without smell or taste, like rain and spring water. On the other hand, the aim of the authorities was the same: to improve health and sanitation as much as possible by supplying ‘safe water’, but the awareness of the Romans was restricted. If necessary, surface water was filtered ^3^, but they were unable to remove invisible but noxious elements like helminths’ eggs.

 In the third paragraph (‘Many pathogens … catastrophs’) Mulder refers to a tripartite division of waters (‘Already… irrigation’), which would be mentioned by Frontinus. I am not aware of any mention of a tripartite division of good, intermediate and bad water in Frontinus; a reference would be welcome. In ch. 11 of *De aquis*, Frontinus describes how the Aqua Alsietina, supplying water of less quality, was constructed for the Naumachia, a pond for reenacting sea-battles; this aqueduct was not connected with the other aqueducts, being situated on the right bank of the Tiber river. This water was not used, in the first instance, for consumption; surplus of the Naumachia was used for the irrigation of private gardens ^4^. In the chapters 88-93, Frontinus criticizes the indiscriminate use of water from aqueducts for any purpose (for instance, the use of clear drinking water for fulleries), and pleads for separating all aqueducts; some aqueducts (like Marcia) containing excellent water have to supply drinking water, other ones (like Anio Vetus) for other purposes ^5^.

 A small correction in this paragraph: for ‘terms’ read ‘thermae’ – which, however, is already covered by ‘baths’. I advise to delete this word.

 Conclusion: Mulder discusses the influence of sanitation on hygiene and health in Antiquity. He disagrees with Mitchell, who states that the improvement of sanitation did not improve human health very much. Bodily hygiene increased by the use of bathhouses, but the number of helminths did not diminish due to the growing use of faeces as fertilizer. Mulder rightly argues that one should not too readily conclude that the development of sanitation did not contribute to the health of the population significantly; more research comparing areas both within and outside the Roman Empire is certainly required. Please add references of Frontinus.

I have read this submission. I believe that I have an appropriate level of expertise to confirm that it is of an acceptable scientific standard, however I have significant reservations, as outlined above.

## References

[1] van TilburgC: Streets and Streams: Health conditions and city planning. *Leiden: Primavera Pers*.2015;100 fn. 59

[2] van TilburgC: Greek and Roman Ideas about Healthy Drinking-water in Theory and Practice. *Eä Journal*.2013;5:1-30

[3] van TilburgC: Streets and Streams: Health conditions and city planning. *Leiden: Primavera Pers*.2015;101

[4] FrontinoSJ: Frontinus, the stratagems and the aqueducts of Rome, translation of C.E. Bennett. *The Loeb classical library*.1925;353

[5] FrontinoSJ: Frontinus, the stratagems and the aqueducts of Rome, translation of C.E. Bennett. *The Loeb classical library*.1925;421

# Response

**Published:** 2017-08-08

**Authors:** Christian Mulder

**Affiliations:** RIVM, Netherlands

I am grateful for the comments by van Tilburg, with whom I agree that present-day microbiological criteria are inapplicable to ancient sanitation systems. However, it seems to me also relevant to state that by changing the (Hellenistic) cisterns – open streaming water cyclus into a distribution system based on streaming and flushing in pipes, the Romans provided globally the very first improvement in human health and public sanitation. Even Plinius [as mentioned by van Tilburg (2013) in the Eä Journal] reported that cistern water had to be regarded as unhealthy, clarifying the relevance of streaming waters. On the other hand, there was surely a huge difference in the water quality across the Roman Empire, with some severe historical cases of poisoning and heavy metal pollution around the borders. But the few citations by van Tilburg are neither examples of human contamination by intestinal parasites nor examples of incorrect water distribution [please read a.o. P. Pace (1983) Gli Acquedotti di Roma, pp. 330, and Rodgers’ English translation of Frontinus’ *De Aquaeductu Urbis Romae*, *Liber Alter*]. As a matter of fact, there has not been during the past a significant growing use of human faeces as fertilizer, but simply an increase of manure application as shown in hundreds of palynological records by the kind of coprophilous dung fungi recorded across Europe and in some sites of Africa. Hence, also the suggested growing use of manure did not occur in urban ecosystems (and van Tilburg correctly wrote “Faeces was used as a fertilizer in the countryside”), although in some countries this unfortunate coexistence of cattle, dogs and people remains an everyday reality. In any case, access to piped water is considered central for a prevention of soil-transmitted nematodes. This reinforces my plea for a better comparison of areas within and outside the Roman Empire and I am very happy to see that also van Tilburg sees it as required. Such pathogens are in fact known from proto- and prehistoric Europe in the Neolithic and during the La Tène culture, and after the decline of the Roman Empire in the post-medieval period as well. Hence, the mechanistic factor beyond the occurrence of helminth eggs is not the Roman water infrastructure (as suggested by archaeologists like P.D. Mitchell), but something else that has still to be discovered (Scarce vegetation cover? Poor-quality housing? Domestic behavior?). Summarizing, these pathogenic nematodes have been widely reported in archaeobotanical and palynological literature because they are intrinsically correlated to human settlements, and not to sanitation infrastructures which only reduce the risk to get in contact with such outbreak sources.

# Peer review: Referee response for version 1

**DOI:** 10.5256/f1000research.12694.r23627

**Published:** 2017-07-17

**Authors:** Karl J. Reinhard

**Affiliations:** 1 School of Natural Resources, University of Nebraska, Lincoln, NE, USA

Christian Mulder’s  “Pathogenic helminths in the past: Much ado about nothing” reveals some basic problems with the research presented by Piers Mitchell in  “Human parasites in the Roman World: health consequences of conquering an empire. Parasitology”. The critique recognizes that the conclusions of the study are overblown and points to errors in methods and theory. I read Mitchell’s work and concur with Mulder. Mitchell does not approach the problem from the perspective of the science of archaeology nor the science of parasitology. Mulder suggests that Mitchell did not control for false parasitism through spread of eggs through the environment. In the science of archaeology, as applied to parasitology, concentrations of parasite eggs per ml or gr are calculated by researchers. These calculations document the distribution of eggs through strata within pits and across ancient village landscapes. This leads to statistical identification of transmission points. When these archaeological egg accumulations are verified as fecal deposits via ancillary pollen and seed analysis, then fecal contamination “hot spots” are defined. Dating of these points can lead to very solid information about emergence and control of geohelminths. This has been demonstrated archaeologically by the references of Fisher and Trigg’s work below. Secondly, the geohelminth life cycle of ascarids seems to be misunderstood by Mitchell who asserted that people could have been infected in Roman baths. 

 I have a paper that has just been published addressing rigor in archaeological parasitology. This highlights the sort of issues such as false parasitism noted by Mulder.

 I believe that Mulder is spot on with regard to Mitchell’s assertion. I strongly recommend this work for indexing and I would hope that Mulder includes references to the work by Fisher’s team and Trigg’s team. The work by Trigg is in press and can be obtained via Heather Trigg or Steve Mrozowski. 

 Useful References:

- Fisher et al. (2007) ^1^
- Trigg et al. (2017) ^2^
- Reinhard (2017) ^3^

I have read this submission. I believe that I have an appropriate level of expertise to confirm that it is of an acceptable scientific standard.

## References

[1] FisherCLReinhardKKirkMDiVirgiloJ: Privies and Parasites: The Archaeology of Health Conditions in Albany, New York. *Historical Archaeology*.2007;172-197

[2] TriggHBJacobucciSAMrozowskiSASteinbergJM: Archaeological Parasites as Indictors of Environmental Change in Urbanizing Landscapes: Implications for Health and Social Status. *American Antiquity*.2017;1-19

[3] ReinhardK: Reestablishing rigor in archaeological parasitology. *International Journal of Paleopathology*.2017; 10.1016/j.ijpp.2017.06.002 10.1016/j.ijpp.2017.06.002 29198394
